# Editorial: The development and plasticity of myeloid immunity in the lung

**DOI:** 10.3389/fimmu.2023.1332852

**Published:** 2023-11-16

**Authors:** Sjoerd T. T. Schetters, Floris Dammeijer, Christophe J. Desmet

**Affiliations:** ^1^ Center for Inflammation Research, Flanders Institute for Biotechnology, Ghent, Belgium; ^2^ Department of Internal Medicine and Pediatrics, Ghent University, Belgium, Ghent, Belgium; ^3^ Department of Pulmonary Medicine, Erasmus Medical Center, Rotterdam, Netherlands; ^4^ Laboratory of Tumor Immunology, Department of Medical Oncology, Cancer Institute, Erasmus Medical Center, Rotterdam, Netherlands; ^5^ Laboratory of Cellular and Molecular Immunology, GIGA Inflammation, Infection and Immunity, GIGA Institute, University of Liège, Liège, Belgium; ^6^ Laboratory of Pneumology, GIGA Inflammation, Infection and Immunity, GIGA Institute, University of Liège, Liège, Belgium

**Keywords:** alveolar macrophage, innate immunity, lung immune cells, myeloid plasticity, asthma, ARDS (acute respiratory disease syndrome), respiratory viral and bacterial infections

The immune system of the respiratory tract comprises a wide variety of myeloid cells, including macrophages, monocytes, mast cells, basophils, neutrophils and eosinophils. Far from their historic view as invariant terminal effector cells, myeloid cells can respond to different types of environmental insults through plastic adaptation of their development and function. In the respiratory tract, no other cell better illustrates this plasticity than the lung macrophages (1). For example, alveolar macrophages (AMs) are long-lived motile cells in the alveolar space, functioning as sentinels that scavenge innocuous airborne antigens or potentially harmful pathogens in the steady-state. In doing so, AMs are critical in maintaining homeostasis and preventing unnecessary inflammation in the delicate airways.

In this Research Topic, Sabatel and Bureau argue that this maintenance is an active and tightly controlled regulatory mechanism that counteracts immunostimulatory processes. AMs and lung-resident interstitial macrophages have been shown to differentially mediate immune tolerance to harmless antigens, while also being able to launch full-blown inflammation when the homeostatic threshold has been exceeded. Respiratory viral infections and allergic inflammation are characterized by recruitment of inflammatory granulocytes like eosinophils and neutrophils, and may even result in complete loss of AMs. Indeed, Feo-Lucas et al. elegantly show that allergic inflammation induced by exposure to respiratory house dust mite (HDM) leads to loss of tissue-resident AMs and the consecutive replacement of long-lived monocyte-derived AMs. This was accompanied by extensive alveolar damage, resulting in reduced gas exchange and surfactant dysfunction. In a similar effort, Draijer et al. investigate the temporal changes in AMs and IMs upon HDM exposure. AMs seemed to proliferate and polarize to a type 2 phenotype upon HDM re-challenge, expressing YM-1.

The inflammatory insults induced by viral infections or allergic inflammation are not without their long-term consequences, as is extensively summarized by (Rodriguez-Rodriguez et al.). They illustrate how the function of long-lived macrophages, including AMs, is dictated by origin, location and previous immunological experience. Future research will need to take these factors into account, since the exact differential role of infiltrating monocytes and tissue-resident macrophages remains an intense matter of debate (2–4). Especially with research aiming to reprogram resident macrophages or infiltrating monocytes as a therapeutic option, it is critical to understand their origin and spatio-temporal control (5–7).

The capacity of AMs to scavenge pathogens or dead cells has been suggested to be their main function in homeostasis and a failure to do so may result in pathology. In this regard, Slimmen et al. provide an interesting description of AMs derived from fresh alveolar lavages from a cohort of pediatric cystic fibrosis patients. They show that AMs from CF patients express lower levels of phagocytic receptors, possibly leading to inefficient clearance of debris or dead neutrophils in CF pathology. Indeed, the ability of AMs to cloak the alveolar space from pathogenic signals prevents neutrophilia (8).

Finally, in this Research Topic, Wang et al. review the current literature on neutrophil (dys)function during acute respiratory distress syndrome (ARDS), a pathological state of respiratory failure associated with severe COVID infection and bacterial sepsis. It is worth noting that although neutrophils are considered short-lived inflammatory cells, in chronic pathological conditions (like asthma, COVID-19 or brain tumors) neutrophils can survive in tissues for extended periods of time and assume a variety of persistent phenotypes (9).

The spatio-temporal control of the myeloid innate immune system remains a prolific topic of investigation in airway diseases. Future research aimed at understanding its development from (early-life) progenitors in interaction with the local niche will undoubtedly further our understanding and provide new avenues for therapeutic interventions.

## Author contributions

SS: Writing – original draft, Writing – review & editing. FD: Writing – review & editing. CD: Writing – original draft, Writing – review & editing.
